# Real-Time Measurements of the Redox States of *c*-Type Cytochromes in Electroactive Biofilms: A Confocal Resonance Raman Microscopy Study

**DOI:** 10.1371/journal.pone.0089918

**Published:** 2014-02-25

**Authors:** Bernardino Virdis, Diego Millo, Bogdan C. Donose, Damien J. Batstone

**Affiliations:** 1 Centre for Microbial Electrosynthesis (CEMES), The University of Queensland, Brisbane, Queensland, Australia; 2 Advanced Water Management Centre (AWMC), The University of Queensland, Brisbane, Queensland, Australia; 3 Biomolecular Spectroscopy/LaserLaB Amsterdam, Vrije Universiteit Amsterdam, Amsterdam, The Netherlands; Russian Academy of Sciences, Institute for Biological Instrumentation, Russian Federation

## Abstract

Confocal Resonance Raman Microscopy (CRRM) was used to probe variations of redox state of *c*-type cytochromes embedded in living mixed-culture electroactive biofilms exposed to different electrode polarizations, under potentiostatic and potentiodynamic conditions. In the absence of the metabolic substrate acetate, the redox state of cytochromes followed the application of reducing and oxidizing electrode potentials. Real-time monitoring of the redox state of cytochromes during cyclic voltammetry (CV) in a potential window where cytochromes reduction occurs, evidenced a measurable time delay between the oxidation of redox cofactors probed by CV at the electrode interface, and oxidation of distal cytochromes probed by CRRM. This delay was used to tentatively estimate the diffusivity of electrons through the biofilm. In the presence of acetate, the resonance Raman spectra of young (10 days, *j* = 208±49 µA cm^−2^) and mature (57 days, *j* = 267±73 µA cm^−2^) biofilms show that cytochromes remained oxidized homogeneously even at layers as far as 70 µm from the electrode, implying the existence of slow metabolic kinetics that do not result in the formation of a redox gradient inside the biofilm during anode respiration. However, old biofilms (80 days, *j* = 190±37 µA cm^−2^) with thickness above 100 µm were characterized by reduced catalytic activity compared to the previous developing stages. The cytochromes in these biofilm were mainly in the reduced redox state, showing that only aged mixed-culture biofilms accumulate electrons during anode respiration. These results differ substantially from recent observations in pure *Geobacter sulfurreducens* electroactive biofilms, in which accumulation of reduced cytochromes is already observed in thinner biofilms, thus suggesting different bottlenecks in current production for mixed-culture and *G. sulfurreducens* biofilms.

## Introduction

The capability of certain microorganisms to exchange electrons with solid surfaces has been subject of intense research in recent years. This process, referred to as extracellular electron transfer, plays a crucial role in the oxidation of organic matter in natural environments such as sediments, as well as in novel technologies such as bioelectrochemical systems, where electrochemically active microbes enable the production of electric energy from the oxidation of waste organic matter in microbial fuel cells, or the production of hydrogen and other chemicals in microbial electrolysis cells. Electricity-driven biosynthesis has become attractive due to the potential to replace agricultural derived feeds, such as sugars and starch, with cheap electricity and oxidized carbon sources, such as CO_2_, for the sustainable production of fuels and bulk chemicals [1].

Whereas bioelectrochemical systems can operate using planktonic cells floating freely in the culturing medium and using mediating redox molecules to facilitate electron transfer, electrochemically active organisms arranged to form biofilms (herein referred to as electroactive biofilms), appear as the most promising. In fact, due to their ability to transfer electrons directly through a conductive biofilm matrix, they can achieve higher kinetics rates than systems relying solely upon diffusion of redox mediators [2], [3]. Although substantial progress has been achieved in regards to optimization of reactor design [4], power output is still not at a level adequate to make practical applications commercially viable, in spite of strong economic and scientific drivers [1]. A large part of this is due to insufficient knowledge in the fundamentals of electron transfer mechanisms and energy management in electroactive biofilms [1]. Two main mechanisms have been proposed to explain electron conduction in multilayered biofilms of *Geobacter sulfurreducens*, an important model organisms extensively studied in bioelectrochemical research: *superexchange* conductivity, according to which electrons are transferred between heme groups of adjacent cytochromes dispersed in the exocellular polymeric matrix forming the biofilm [5], and *metallic-like* conduction through *nanowires*, whereby electric conductivity is due to the conducting properties of the pilA protein composing the filaments [6]. In this case, multiheme cytochromes are suggested to mediate the electron transfer from the nanowire to the electrode. Regardless of the actual mechanism, the intrinsic resistance of the conductive matrix controls the rate of the electron transfer and generates a measurable redox gradient across the biofilm [7]. However, the amplitude of this redox gradient would be considerably different for the two proposed mechanisms. Biofilm conductivity enabled by metallic-like mechanism is expected to be higher than that of a system where electron transfer occurs via the electron hopping mechanism [6]. In fact, in a system governed by metallic-like conduction the redox variations across long distances are expected to be hardly measurable with available technologies [8], while the accumulation of reduced cytochromes in biofilms of *G. sulfurreducens* have been reported by Robuschi *et al*., consistently with the presence of conduction pathways at higher resistance [9].

The ability to map the redox state in biofilms while actively performing electron transfer is of particular interest in microbial bioelectrochemical systems research, as it allows probing the redox properties of biofilms also at defined distances from the electrode, thus complementing the information given by other electrochemical methods such as cyclic voltammetry (CV). Techniques based on absorption, such as IR or UV/Vis spectroscopy, or scattering, such as Raman spectroscopy, allow the identification of molecules based on their spectral fingerprints. Resonance Raman (RR) spectroscopy exploits the enhancement of the spectral intensity that is achieved when the frequency of the excitation laser line is close to an electronic transition of a chromophore present in the sample, *e.g.*, the heme group of a redox protein [10]. High spatial resolution can be achieved by combining the capabilities of Raman spectroscopy with the spatial resolution of confocal laser scanning microscopy. Using this approach, we recently demonstrated that Confocal Resonance Raman Microscopy (CRRM) can be used to resolve the spatial distribution of outer membrane cytochromes *in vivo*, without impacting on the catalytic activity of *Geobacter* enriched biofilms [11].

In this study, we provide additional evidence of the involvement of *c*-type cytochromes in electrode-biofilm interaction in mixed-culture electroactive biofilms. This includes analysing the Resonant Raman spectra in detail to assess the redox state of outer membrane and periplasmic cytochromes obtained from living electroactive biofilms exposed to reducing or oxidizing electrode polarizations, under both turnover and nonturnover conditions (*i.e.*, in the presence and absence of the metabolic substrate, respectively), at different biofilm locations, and at different developing stages.

## Results and Discussion

### Electrochemical activity of mixed-culture biofilms

**Figure 1:**
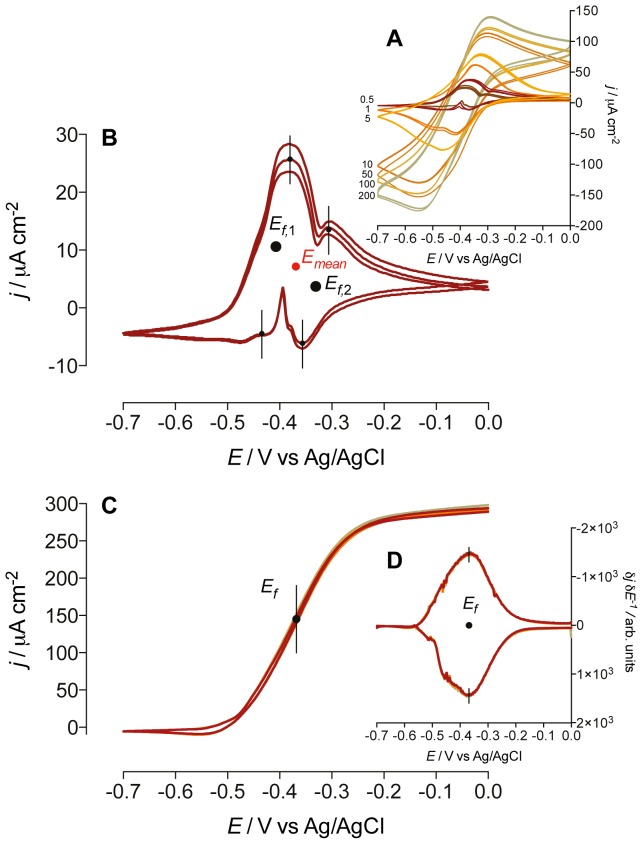
Cyclic voltammograms (CVs) obtained from the electroactive biofilms. A) Nonturnover CVs recorded in acetate-depleted medium at scan rates ranging from 0.5 to 200 mV s^−1^ (as indicated). B) Nonturnover CV recorded at the scan rate of 0.5 mV s^−1^. The two redox couples *E_f,1_* and *E_f,2_* are centered at the formal potentials of −407 mV and −331 mV, respectively. *E_mean_*, centered at −369 mV, indicates the arithmetic average of *E_f,1_* and *E_f,2_*. C) CVs of an electroactive biofilm metabolizing acetate (*i.e.*, turnover conditions), recorded at a scan rate of 0.5 mV s^−1^. D) First derivatives of the turnover voltammetric curve performed at 0.5 mV s^−1^. *E_f_* represents the putative electron-transfer site, centered at −368 mV.

Electroactive biofilms were incubated at a fixed potential of 0 V vs. Ag/AgCl on glassy carbon electrodes using 20 mM sodium acetate as metabolic substrate. The voltammetric behavior of the biofilms was frequently monitored under turnover and nonturnover conditions. Although voltammograms recorded at scan rates above 5 mV s^−1^ in acetate-depleted medium display one redox couple (Figure 1A), CVs performed at the lowest scan rate of 0.5 mV s^−1^ reveal two redox couples, namely *E_f,_*_1_ and *E_f,_*_2_, centered at formal potentials of −407 mV and −331 mV (Figure 1B). It is important to note that all peaks were retained even after the medium was replenished with fresh sterile solution, indicating that the voltammetric signals were all ascribed to redox compounds embedded inside the biofilm (data not shown). Figure 1C shows a voltammogram recorded at the scan rate of 0.5 mV s^−1^ under turnover conditions. The CV traces are characterized by the sigmoidal shape typical of electrocatalytic activity due to substrate oxidation. First derivative analysis of the voltammetric curves in Figure 1D reveals two maxima (hereby depicted as one maximum and one minimum for the voltammetric scan in the anodic and the cathodic direction, respectively) in correspondence to the inflection points of the oxidative and the reductive sweep, respectively. This indicates one apparent redox center *E_f_* with formal potential of −368 mV, which is very close to the arithmetic average of the redox couples *E_f,_*_1_ and *E_f,_*_2_ (*E_mean_* = −369 mV indicated in Figure 1B), suggesting the involvement of both redox couples in the catalytic current. Noteworthy, the overall shape and peak positions of the CV traces of our mixed-culture biofilms are very similar to those obtained for biofilms of pure *G. sulfurreducens* cultures [12]–[14]. This similarity is supported by pyrosequencing analysis on DNA samples extracted from similar mixed-culture biofilms showing a high enrichment of *Geobacteraceae* (>85% of the sequences clustered 100% with an uncultured *Geobacter* strain NS1) [11]. Despite these similarities, we will show how the electrical and structural properties of mixed-cultures may differ substantially from those reported for pure *G. sulfurreducens* biofilms.

### Resonance Raman scattering of biofilms under potentiostatic control in nonturnover conditions

**Figure 2:**
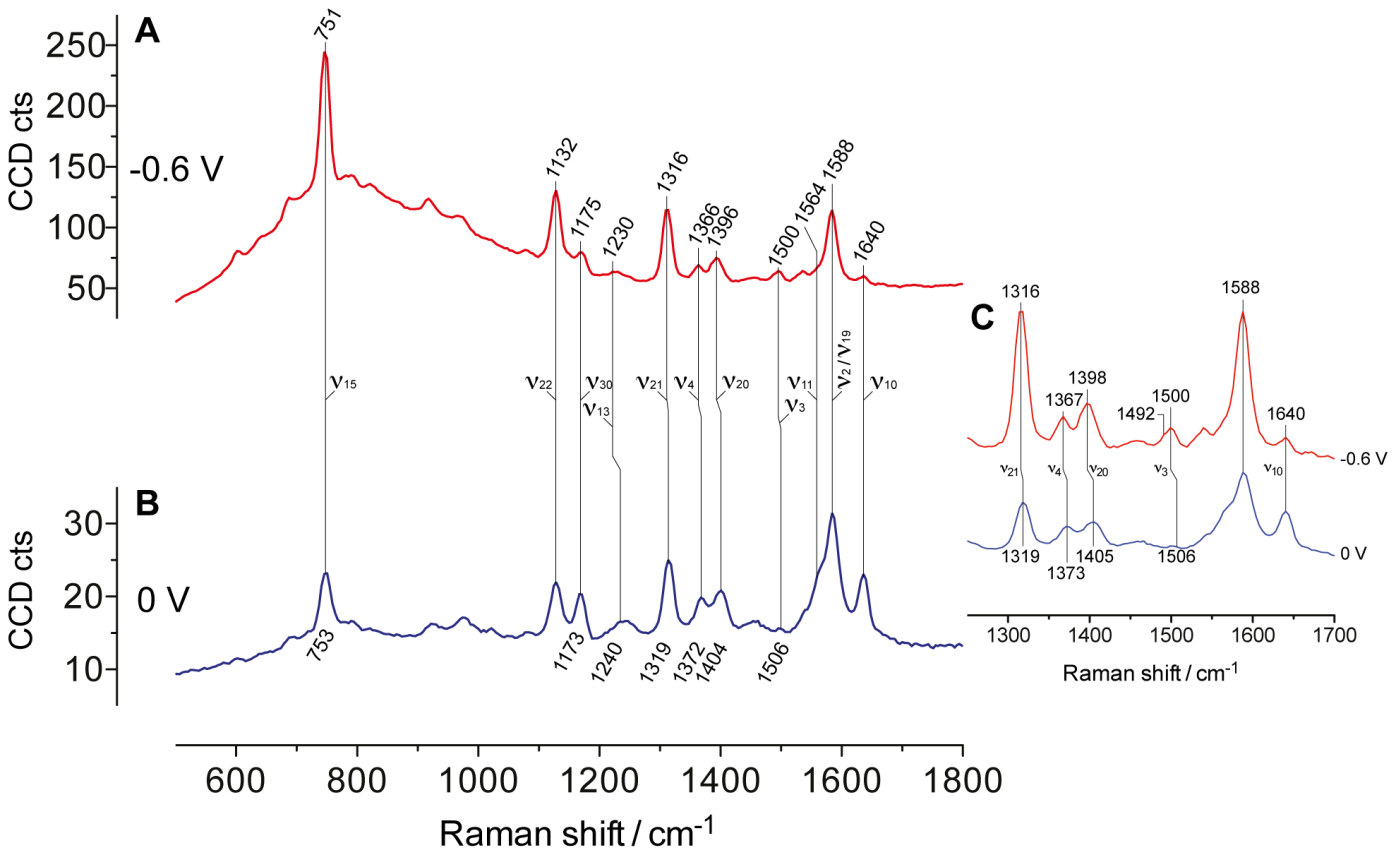
Typical Resonance Raman spectra of electroactive biofilm in the marker region between 550 and 1800^−1^ obtained under different electrode polarizations in the absence of metabolic electron donor. A) RR spectra obtained with the working electrode poised at −0.6 V vs. Ag/AgCl. B) RR spectra obtained with the working electrode poised at 0 V vs. Ag/AgCl. C) Detail on RR spectra in the region 1250–1700 cm^−1^. For clarity, the intensity of the spectra acquired at 0 V is multiplied by 2.

Raman spectra of mixed-culture electroactive biofilms measured in acetate-depleted medium with the electrode poised at −0.6 V and 0 V vs. Ag/AgCl exhibit strong resonance scattering signals, dominated by the vibrational bands of *c*-type heme groups of the cytochromes embedded inside the biofilm (Figure 2A and 2B). Details on peak assignment are summarized in Table S1 in Supporting information. The relative intensities of the bands in a resonance Raman spectrum of a heme group vary as a function of the incident wavelength. Whereas wavelengths in resonance with the Soret band of the heme (*e.g.*, the 413 nm laser line) enhance the totally symmetric vibrational modes (*i.e.*, the A_1g_ modes such as the *ν*_4_, *ν*_3_, and *ν*_2_), wavelengths in resonance with the broad *Q* absorption band of the heme – such as the 532 nm laser line used in this study – enhance the non-totally symmetric modes (*i.e.*, the A_2g_, B_1g_, and B_2g_ such as the *ν*_10_) [15]. RR spectra are rich in the mid- (1100–1700 cm^−1^) and low- (600–800 cm^−1^) frequency regions, particularly the modes *ν*_15_ (B_1g_), *ν*_22_ (A_2g_), *ν*_30_ (B_2g_), *ν*_21_ (A_2g_), *ν*_10_ (B_1g_). Characteristic of the low frequency region is the strong enhancement of the pyrrole breathing mode *ν*_15_ at around 750 cm^−1^ observed at both wavelengths. The absence of other vibrational features in this region makes this mode a convenient marker for the presence of *c*-type hemes [16]. The intensity of this band was also suggested to be proportional to the amount of reduced cytochrome *c* in mitochondrial cells [17], [18]. Modes in the high-frequency region (1300–1700 cm^−1^) originates predominantly from C–C and C–N stretching vibrations of the porphyrin and are indicative of the oxidation-, spin-, and coordination-state of the iron atom [19]. These bands, denoted as *ν*_4_, *ν*_20_, *ν*_3_, *ν*_2_, and *ν*_10_, at the applied potential of 0 V are centered at 1372, 1404, 1506, 1588, and 1640 cm^-1^, respectively. Applying the potential of −0.6 V to the electrode leads to a downshift of *ν*_4_, *ν*_20_, and *ν*_3_ to 1366, 1396, and 1500 (a broad band with a shoulder at 1492) cm^−1^, respectively. Remarkably, the position of the *ν*_10_ does not vary although its relative intensity diminishes significantly. These changes are consistent with an oxidized (at 0 V) and a reduced (at −0.6 V) heme group having a six-coordinated iron atom in a low-spin state with two His residues acting as axial ligands of the heme iron atom [13], [20], [21]. Most likely, these heme groups belong to the outer membrane cytochromes and the periplasmic cytochromes wiring the cell metabolism to the electrode [10]. Although the bis-His heme ligation is typical of mixed-culture biofilms dominated by *Geobacter* species [10], [13], pure *G. sulfurreducens* biofilms grown on Indium tin oxide electrodes display a different ligation [9]. In fact, the cited study reports two *ν*_10_ bands at 1620 and 1638 cm^−1^, whose relative intensities vary considerably as a function of the applied potential. While the intensity of the band at 1620 cm^−1^ decreases, the band at 1638 cm^−1^ increases when the electrode potential is varied from −0.65 to 0.05 V [9]. This trend reveals the presence of at least two different six-coordinated low-spin heme states having the His/His and the Met/His axial ligation of the iron atom, as probed by the *ν*_10_ at 1638 and 1620 cm^−1^ respectively [21]. These differences, if confirmed by future experiments, may enable the use of Raman spectroscopy to discriminate between different microbial communities on electrodes.

**Figure 3:**
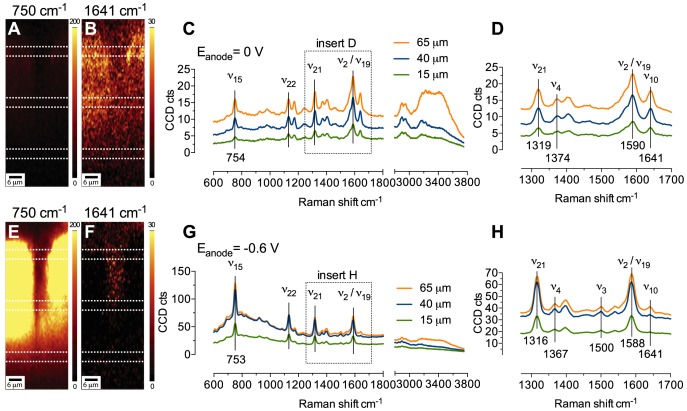
Biofilm sectional images and average RR spectra collected from electroactive biofilm at day 57 since inoculation under nonturnover conditions, with the electrode poised at 0(A, B, C, and D) or at −0.6 V (E, F, G, and H). Sectional maps (A, B, E, and F) are generated by binning the whole spectral information at 750 cm^−1^ (A and E), and at 1641 cm^−1^ (B and F), used here as markers of reduced and oxidized cytochromes, respectively. Each spectrum was averaged within the areas indicated by the dotted lines, representative of three biofilm locations, that is, top, middle, and bottom, respectively at a distance of 65, 40, and 15 µm from the electrode surface (not visible at the bottom of the spectral maps). The biofilm was exposed to the respective potentials for a period of at least 20 minutes to allow complete oxidation or reduction of cyt *c*, as suggested by stabilization of the current profile to very low levels (profiles shown in Figure S2A and B).

The RR spectra reported in Figure 2 were averaged between spectra collected in areas of highest signal intensity over the whole biofilm. However, when spectra were averaged on other biofilm portions, regardless of the distance from the electrode, their spectral features were again consistent with a reduced or an oxidized cytochrome, depending on the applied potential, confirming that the particular redox state was maintained homogenously across the whole biofilm. One of the advantages of the use of a confocal microscope with Raman capability is its capacity to collect spectral information from different focal planes and hence, different sample locations. Figure 3 shows the RR spectra obtained in acetate-depleted medium by averaging the spectral information from biofilm locations at different distances from the electrode surface. The overall biofilm depth in this particular location was about 70 µm and the RR profiles depicted in Figure 3 are averaged from portions centered, respectively, at 15 µm, 40 µm, and 65 µm from the electrode, indicated for simplicity as bottom, middle, and top, respectively. The figure shows that the collected spectra have very similar spectral features to those reported in Figure 2 with the electrode polarized at 0 V and −0.6 V. In particular, the relative intensities and the Raman shifts of the marker modes *ν*_4_, *ν*_3_, and *ν*_10_ are consistent with the presence of oxidized and reduced cytochromes maintaining the six-coordinated low-spin bis-His configuration in both redox states. The overall signal intensity weakens as the signal is collected from deeper portions (this is particularly evident for the traces collected with the electrode poised at −0.6 V). This observation suggests the progressive dissipation of the RR signal due to thicker biofilm portions that is necessary to be transited by light. Photodegradation was negligible in our measurements due to low integration time used (0.2 s) and low laser power at the sample (<5 mW). Regardless of the loss of signal intensity in the deepest regions of the biofilm, the spectral features reported in Figure 3 (relative intensities and band shifts) are consistent with a switch of redox state of cytochromes following the application of oxidizing or reducing electrode potential, and the same redox state is maintained across different biofilm depths. It is worth noting that concluding on the redox state of the cytochromes basing on the intensity of one band only would be misleading and may lead to an underestimation of the real amount of reduced species, especially in the vicinity of the electrode. Therefore, in this study we estimate the redox changes by tracking the variations of the intensities of two bands, specifically *ν*_15_ and *ν*_10_. Importantly, these intensity changes must be accompanied by the expected band shifts that are characteristic for the oxidized and the reduced heme species, such as the shifts in the mid-frequency region (*vide supra*). Figure 3 (panels A, B, E, and F) shows typical sectional images of the biofilms obtained by filtering the whole spectral information collected over a biofilm section for the mode *ν*_15_ at around 750 cm^−1^, and for the mode *ν*_10_ at around 1641 cm^−1^. The glassy carbon electrode sits at the bottom of the images, however, it is not visible in the sections due to the narrow binning used to generate the images (which does not include the peaks at 1352, 1585, and 2700 cm^−1^ characteristic of carbon materials) [22]. The images show position and redox state of the cytochromes over the whole biofilm section probed with changes induced by electrode polarization. Although this information can be deduced from spectral analysis (*vide supra*), the added confocal capability allows the generation of maps depicting the redox state of the cytochromes across large biofilm regions, which can be particularly useful when studying electron transfer through the biofilm. For instance, with the electrode polarized at 0 V, the Raman spectra is dominated by the band at 1641 cm^−1^ (Figure 3B), whereas the band at 750 cm^−1^ appears very dark (Figure 3A), indicating that the redox state of cytochromes is oxidized. The opposite situation arises when the electrode potential is shifted to the negative value of −0.6 V. In this case, the very large increase in intensity achieved with reduced cytochromes results in the very bright map reporting position and intensity of the band at 750 cm^−1^ (Figure 3E). Concurrently, the map depicting the band 1641 cm^−1^ appears very dark due to the lowering of the mode *ν*_10_ upon cytochromes reduction (Figure 3F).

### Real-time monitoring of redox state of cytochromes during cyclic voltammetry in nonturnover

**Figure 4:**
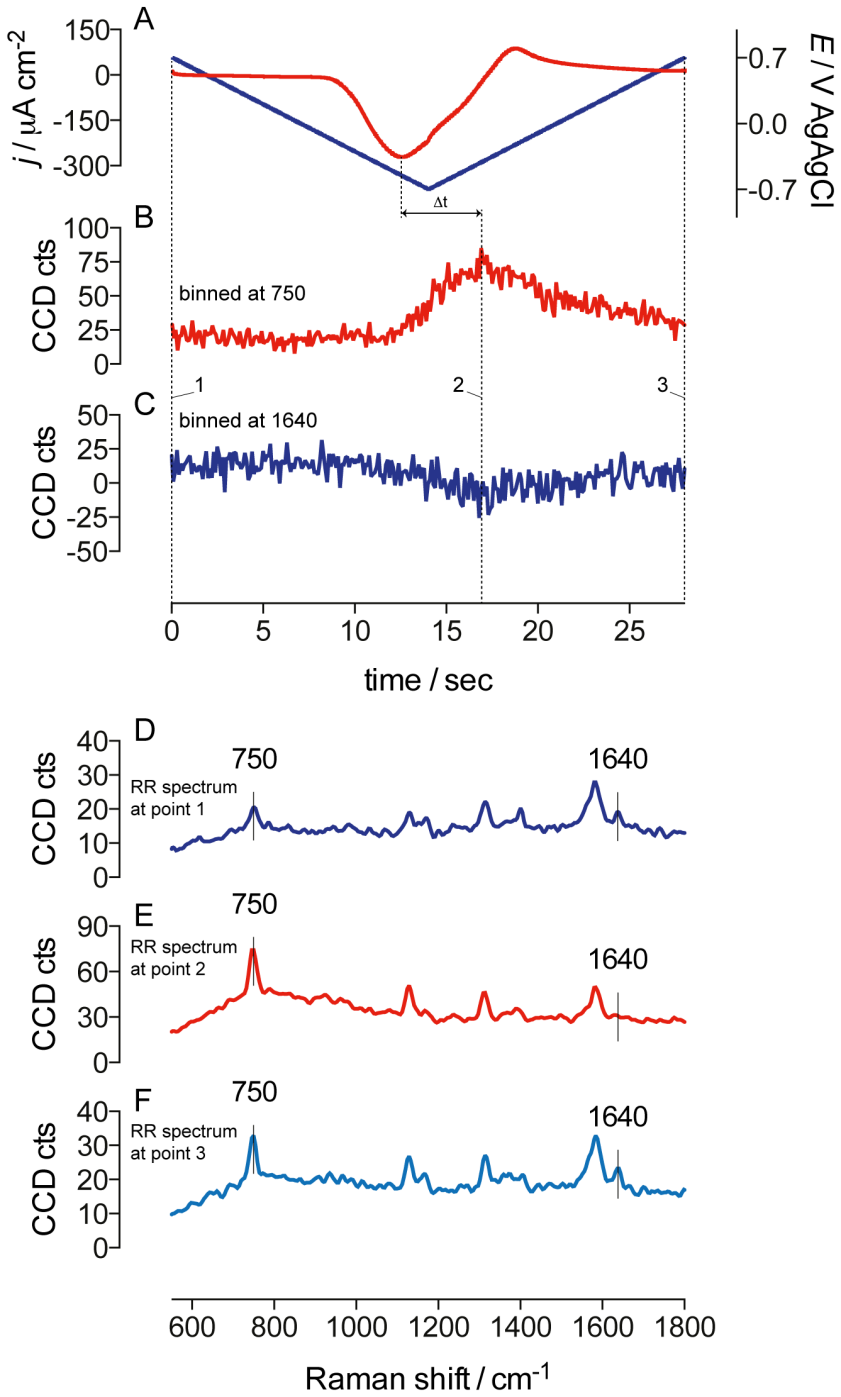
Example of combined voltammetry and RR measurements performed on a mature biofilm in acetate-depleted medium. RR spectra were collected continuously from a fixed portion of the biofilm positioned at approximately 50 µm from the electrode surface. A) current/potential vs. time (scan rate: 100 mV s^−1^). B) time-series of the intensity of the RR spectra binned for the marker mode *ν*_15_ at 750 cm^−1^, and C) the marker mode *ν*_10_ at 1640 cm^−1^. D) RR spectra collected at time point 1 (0 s). E) RR spectra collected at time point 2 (17 s). F) RR spectra collected at time point 3 (28 s).

The ability of CRRM to collect RR spectra using a short accumulation time allows real-time measurements of changes in the redox state of the cytochromes during dynamic variations of the electrode polarization. Figure 4 shows an example of RR observations performed on a single biofilm spot positioned at about 50 µm distance from the electrode in a region of high signal intensity for the cytochromes. During the measurement, the electrode potential was cyclically swept between the two limit potentials of −0.7 V and +0.7 V at the scan rate of 100 mV s^−1^, under nonturnover conditions. Figure 4A depicts the evolution of the nonturnover current density during the test. Similarly to the CV profiles reported in Figure 1, and as expected by nonturnover conditions, the profile of current highlights oxidative (positive current densities *j*) reductive (negative *j*) peaks in correspondence to which cytochromes are oxidized and reduced [23]. RR spectra were continuously recorded during the measurements. Panels D to F in Figure 4 report RR spectra at the specific time points indicated. It is interesting to note the presence of substantial changes in the spectral features ascribed to the RR spectra recorded over time. In particular, Figure 4B and C plot the time variations of the intensity of the marker bands *ν*_15_ and *ν*_10_ at 750 cm^−1^ and 1640 cm^−1^ that are proportional to the amount of a *c*-type heme in the reduced and in the oxidized state, respectively (*vide supra*). The time-series traces show remarkable changes in the spectral intensity ascribed to these two bands. This is consistent to the shift in redox state of the cytochromes following the potential sweeps as suggested by the profile of current density and analysis of the RR spectral features reported in Figure 4D–F. It is worth noting that the maximum intensity value for the marker band *ν*_15_ (and the correspondent minimum intensity value for the marker band *ν*_10_) is not aligned (with respect of the elapsed time) to the reductive peak as shown by the current profile (Figure 4A). This divergence identifies a time delay of 5.4±0.9 s existing between the reduction of the redox cofactors probed by CV and the reduction of the cytochromes probed by RR spectroscopy in the portion observed. This delay indicates that the cytochromes at 50 µm from the electrode do not respond immediately to the applied potential, thus supporting the hypothesis that the voltammetric trace is dominated by the surface-confined cytochromes that are abundant in the vicinity of the electrode [24], whereas remote proteins do not contribute to the CV signal significantly [10]. The reason for the delay resides likely on the finite rate of the long-range electron transfer inside the biofilm, as suggested by others [7]. Our results prove the following: 1) cytochromes detected by CRRM (*i.e.*, outer membrane and periplasmic cytochromes) are electrically connected to the electrode, confirming their involvement in long-range electron transport across electroactive biofilms; 2) CRRM can effectively be used for real-time monitoring of redox variations in electroactive biofilms. Even if the RR approach described hereby does not allow identification of the specific cytochromes embedded inside the biofilm univocally [10], studies on *G. sulfurreducens* have indicated the octaheme OmcZ as the most important cytochrome required in high current producing biofilms [25], [26]. This cytochrome covers a potential window between approximately −600 to −250 mV (vs. Ag/AgCl), which would be in agreement with the formal potential of the redox couple as suggested by the cyclic voltammetry measurements reported in the previous section (estimated to have a formal potential of −368 mV vs. Ag/AgCl); 3) the observed delay between the CV and CRRM signals allows estimating the diffusivity of the electron *D_E_* through the biofilm. Assuming a hopping electron transfer mechanism, *D_E_* may be calculated using the Einstein's approximation for the mean distance of diffusing molecules [27]: 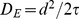, where *d* is the distance travelled by the electrons in a time *τ*. Assuming *d* = 50 µm and *τ* = 5.4 s, the equation gives approximately a *D_E_* of 2.3×10^−6^ cm^2^ s^−1^, which is very similar to the value reported by Bonanni *et al*. for pure *G. sulfurreducens* biofilms [8].

### Redox state of cytochromes under turnover conditions

**Figure 5:**
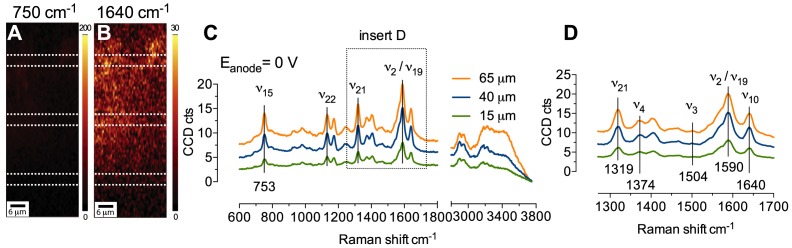
Biofilm sectional images and average RR spectra collected from electroactive biofilm at day 57 since inoculation, obtained after addition of 20 mM sodium acetate with the electrode poised at 0 V. Sectional maps are generated by binning the whole spectral information at (A) 750 cm^−1^, and at (B) 1640 cm^−1^, used here as markers of reduced and oxidized cyt *c*, respectively. Each spectrum was averaged within the areas indicated by the dotted lines, representative of three biofilm locations, that is, top, middle, and bottom, respectively at a distance of 65, 40, and 15 µm from the electrode surface (not visible at the bottom of the spectral sectional maps). Prior recording the RR spectra, the biofilm was exposed to the potential of 0 V vs. Ag/AgCl for 40 minutes to allow stabilization of the current profile (profiles shown in Figure S2C).

Although Figures 2, 3, and 4 above show that in the absence of metabolic substrate the redox state of the cytochromes is controlled by the electrode polarization, recent studies on pure *G. sulfurreducens* biofilms have shown that in the presence of metabolic electron donor, the redox state of cytochromes cannot be fully controlled by the electrode potential. It was observed that during catalytic acetate oxidation, the non-efficient electron transport across large biofilm portions generates a concentration gradient of oxidized cytochromes that is likely to limit the respiration rate of bacterial cells in the outer layers of the biofilm [9], [24], [28]. To identify that this is also the case for our mixed-culture biofilms, we performed RR measurements on electroactive biofilms while exposed to the electrode polarization of 0 V vs. Ag/AgCl, in the presence of 20 mM sodium acetate. Analysis of the spectral features reported in Figure 5 indicates the presence of cytochromes mostly in the oxidized form. This is supported by 1) the position of the *ν*_4_ vibrational mode centered at 1374 cm^−1^, 2) the presence of the intense mode *ν*_10_ at 1640 cm^−1^, and 3) the presence of the mode *ν*_3_ at 1504 cm^−1^. It is worth noting that the relatively low intensities measured for the *ν*_15_ mode under these conditions, are comparable to the intensity measured for the same band under nonturnover conditions with the electrode poised at 0 V, and consistent with oxidized cytochromes (see Figures 2 and 3 for comparison). In addition, similarity between spectra collected from biofilm portions at different distances from the electrode provides no indication of the presence of a redox gradient across the biofilm section examined at this electrode polarization (see panels C and D in Figure 5). Interestingly, the thickness of the analysed biofilms at this stage of growth was approximately 70 µm, hence much larger than the threshold observed in pure *G. sulfurreducens* biofilms beyond which electrons were shown to accumulate as reduced cytochromes [24]. This observation may suggest different electron transfer properties of mixed-culture biofilms with respect to pure *G. sulfurreducens* films. In fact, according to the model describing the electron transfer in biofilms operating in the direct electron transfer mode, the presence of a redox gradient - with the most oxidized cytochromes closer to the electrode - is due to the coupling of the rate-limiting long-range electron transfer between adjacent cytochromes, with the much faster heterogeneous electron transfer across the biofilm/electrode interface [7]. However, the lack of reduced cytochromes even on biofilm layers remote from the electrode indicates that the ratio between heterogeneous and long-range electron transfer rates in mixed-culture biofilms may be lower than in pure *G. sulfurreducens* biofilms. Considering as comparable the values of diffusivity of the electron *D_E_* for pure and mixed-culture biofilms (*vide supra*), then the heterogeneous electron transfer rates in mixed-culture biofilms must be slower than in pure *G. sulfurreducens*. Conversely, slower rate of acetate assimilation, or sluggish electron transport from within the cells to extracellular redox mediators – which would account for lower current density of our biofilm compared to that of *G. sulfurreducens* – may also contribute to explain the lack of redox gradient in the mixed-culture biofilms reported here. Admittedly, the lack of substantial information on the rates of the above-mentioned processes in mixed-culture biofilms does not allow being conclusive on this aspect. Importantly, our experimental approach does not exclude the presence of conducting nanowires, or alternative electron transfer mechanisms, perhaps promoted by different redox molecules and/or microorganisms that co-exist with electron transfer via cytochromes and may play an important role under different growth conditions.

**Figure 6:**
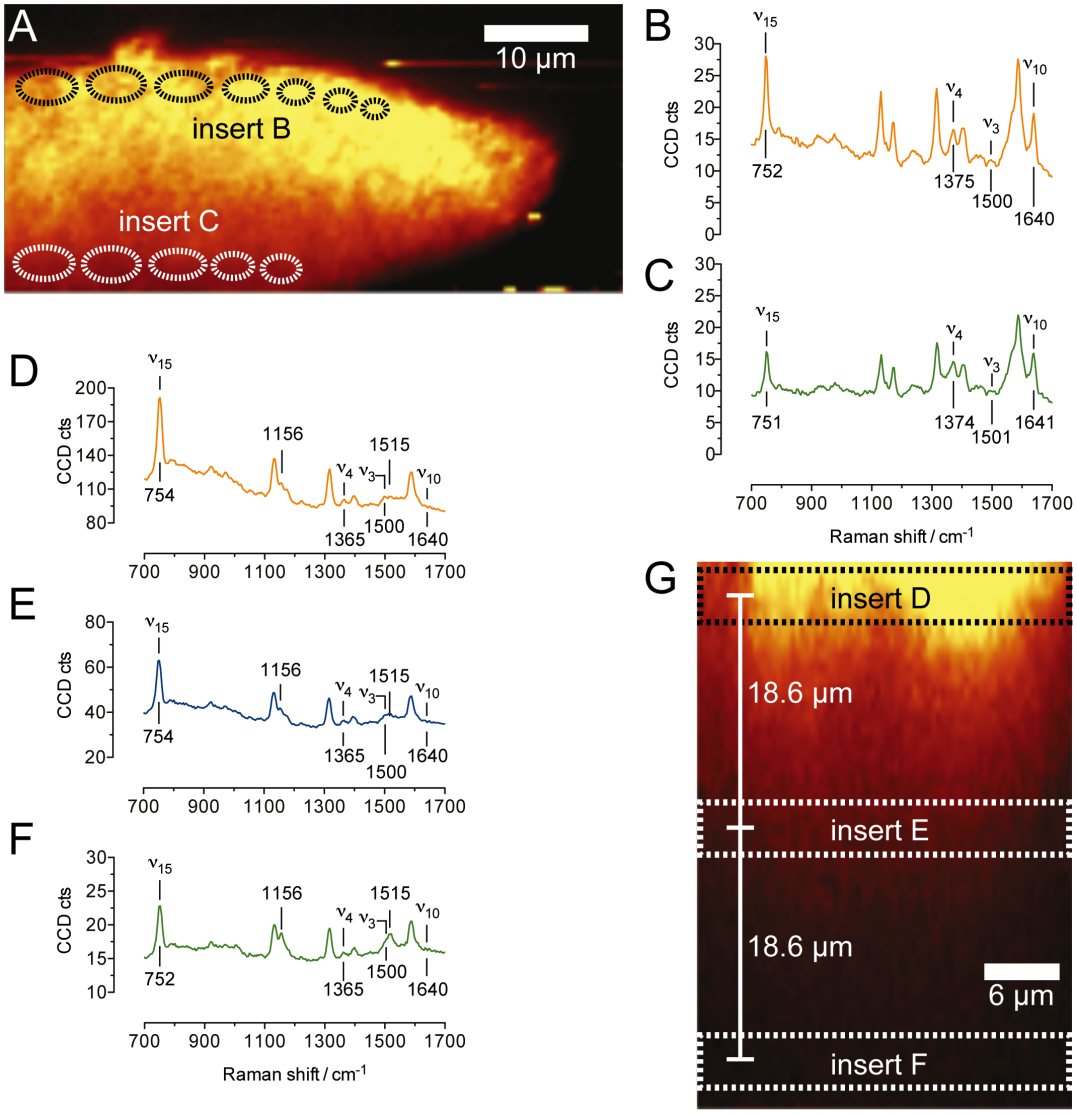
Raman sectional images and RR spectra averaged at different biofilm locations (as indicated by the dashed lines in the sectional maps) obtained from electroactive biofilms at different developing stages, grown at the electrode potential of 0 V vs. Ag/AgCl in the presence of 20 mM sodium acetate. Panels A, B, and C refer to a 10 days old biofilm with thickness <20 µm. Panels D, E, F, and G refer to an 80 days old biofilm with thickness >100 µm. Sectional maps are generated by binning over whole spectral information between 600 and 1800 cm^−1^. Note that only the lower section of the 80 days old biofilm is shown in panel G. The biofilm thickness was in fact too large to be measured in its entirety in one single confocal measurement.

The effect of biofilm aging on the oxidation states of the cytochromes across the films was also addressed. Figure 6 shows RR spectra and sectional maps generated at day 10 after inoculation, when the biofilm thickness was about 20 µm and showed the open structures typical of growing biofilms, probably resulting from the expansion of smaller colonies, which eventually evolved in the pillar geometries as seen in more mature biofilms (see for instance Figure 3). With the electrode polarized at 0 V, the RR spectra show that also in this case the cytochromes are oxidized at any distance from the electrode (Figure 6B and C), which is not surprising given the lower thickness of the films at this developing stage, similarly to what has been observed for *G. sulfurreducens* biofilms [24]. However, when we repeated the measurement on the same biofilm at day 80 we observed cytochromes mostly in the reduced form (Figure 6D–F). This is supported by 1) the absence of the mode *ν*_10_ at around 1640 cm^−1^, and 2) the position of the mode *ν*_4_ at 1365 cm^−1^, in all the three spectra averaged from three different biofilm depths. Figure 6G shows only a biofilm portion of approximately 42 µm from the electrode, while the total biofilm thickness was above 100 µm. Unfortunately, a full section could not be recorded at once because of the z piezo-crystal movement limit. Nevertheless, spectra collected from upper portions of the biofilm (not included in Figure 6) confirmed that this redox state was maintained across the whole section tested. It is important to note that the observed accumulation of reduced cytochromes in later stage biofilms coincide with lowest catalytic activity of the biofilms. The highest current density values for the three replicates as per chronoamperometric profiles (averaged between the three replicates, data not shown) were 208±49 µA cm^−2^ at day 10, 267±73 µA cm^−2^ at day 57, and 190±37 µA cm^−2^ at day 80, which indicates a loss of electrocatalytic activity at the later stages of development. This observation is also corroborated by CV measurements performed at the three developing stages and reported in Supporting information (Figure S1). The decline in the maximal current production and the lack of oxidized cytochromes in the vicinity of the electrode is consistent with a diminished ability to promote an efficient heterogeneous electron transfer. In fact, the presence of reduced cytochromes in aged biofilms indicates that electrons obtained from acetate are stored on the cytochromes, thus ruling out acetate assimilation and electron transport from within cells to extracellular redox mediators as possible rate-limiting steps in this case.

Along with the heme marker bands, old biofilms display two additional bands at 1515 and 1156 cm^−1^ that were not present in younger biofilms. These bands are assigned to β-carotene [29]. Zheng *et al*. recently correlated β-carotene content and aging of *Rhodococcus* sp. SD-74 biofilms [30]. Microorganisms are known to accumulate several types of carotenoids in response to various environmental pressures, including exposure to light, temperature variation, and redox stresses [31]. At this stage, we cannot be assertive on the causes that have triggered the production of β-carotene in our systems. In our opinion, this finding will contribute to stimulate the use of CRRM to detect biomolecules that are different from the heme and may play biologically relevant functions in electroactive biofilms.

## Conclusions

In this study we used Confocal Resonance Raman Microscopy (CRRM) in combination with various electrochemical techniques to investigate the redox properties of mixed-culture electroactive biofilms grown on glassy carbon electrodes. To monitor the redox molecules promoting the electron transfer processes inside the biofilm in real-time, we performed for the first time CRRM measurements in the time-resolved mode. Our results can be summarized as follows:

1. The RR spectra of the biofilm were dominated by the vibrational modes of the heme groups of *c*-type cytochromes linking the metabolism of the microbial cell to the electrode. In the absence of metabolic electron donor, the oxidation state of the cytochromes is entirely controlled by the applied potential through the whole section of the biofilm, in a way that also cytochromes distant from the electrode are electrically wired to it.
2. The central Fe atom of the heme groups is six-coordinated, low-spin, and has two His residues acting as axial ligands. Although this axial ligation is typical of mixed-culture biofilms dominated by the electrochemical signature of *Geobacter* species [10], [13], it differs substantially from the Met/His ligation detected by others in pure *G. sulfurreducens* biofilms on Indium tin oxide electrodes [9]. Moreover, while mixed-culture biofilms at later growth stages present bands associated to β-carotene, similar features have not been reported for pure *G. sulfurreducens* biofilms yet. Although their impact on the redox properties of the biofilm is still unknown, to our knowledge this is the first report pointing out structural differences between mixed and pure culture biofilms.
3. RR spectra obtained during dynamic potential sweeps displayed an appreciable delay of 5.4±0.9 s between the applied potential and the corresponding variation of redox state of the cytochromes at a defined distance from the electrode, allowing to tentatively estimate the diffusivity of electrons through the biofilm *D_E_* as equal to approximately 2.3×10^−6^ cm^2^ s^−1^.
4. Experiments done in the presence of metabolic substrate show that despite the outstanding similarities in the voltammetric traces, the bottleneck of current generation in pure *G. sulfurreducens* and mixed-culture biofilms is different. In fact, our data show that mixed-culture biofilms with thickness up to 70 µm do not show electron accumulation – detectable as cytochrome reduction – and the consequent formation of a redox gradient across the biofilm. However, electron accumulation was observed on aged biofilms after 80 days of incubation, characterized by larger thickness (>100 µm) and diminished electrocatalytic current densities. These findings suggest that the strategies adopted by mixed-culture biofilms to control the electron flow through the biofilm and to the electrode may differ substantially from those reported for pure *G. sulfurreducens*.

## Materials and Methods

### Microbial inoculum and biofilms development

Electrochemically active biofilms were grown on sealed single-chambered reactors using a three-electrode configuration consisting of a 10×20×2 mm glassy carbon plate (SIGRADUR®, HTW Hochtemperatur-Werkstoffe GmbH, Germany) serving as working electrode (anode), a Ti mesh serving as counter electrode, and a Ag/AgCl reference electrode in 3 M KCl (MF-2052, Basi, USA). External connection of the glassy carbon plates was obtained by gluing insulated copper wires on the backside of the plates using conductive glue (Leitsilber 200, Pelco International, USA). Water-resistant, non-toxic, epoxy adhesive was spread on the back and lateral surfaces of the glassy carbon plates to provide electric insulation and mechanical strength to the electrode/wire system, leaving an effective (exposed) surface area of 2 cm^2^. The electrodes were successively polished with alumina slurry of different particle size (1 µm, 0.3 µm and 0.05 µm), rinsed and sonicated in reverse osmosis water after each polishing step. The electrodes were immersed in 400 mL sterile anaerobic media (purged with nitrogen for at least 30 min to ensure anoxic conditions) and the bioelectrochemical systems were operated at 35°C using a water bath. The growth medium consisted of autoclaved deionized water containing: Na_2_HPO_4_ (6.0 g L^−1^), KH_2_PO_4_ (3.0 g L^−1^), NH_4_Cl (0.1 g L^−1^), NaCl (0.5 g L^−1^), MgSO_4_·7H_2_O (0.1 g L^−1^), CaCl_2_·2H_2_O (0.015 g L^−1^), trace elements solution (1 mL, composition in Lu *et al*.) [32], and sodium acetate CH_3_COONa (1.64 g L^−1^, equal to 20 mM) as the metabolic substrate.

Primary biofilms were formed using domestic wastewater from a local wet well as inoculum (5 mL in 400 mL) and incubated in a sealed bioelectrochemical cell using a carbon rod (Morgan AM&T, Australia) as working electrode, poised at 0 V vs. Ag/AgCl using a potentiostat (Potentiostat/Galvanostat VMP3, BioLogic Science Instruments, France), to promote and monitor biofilm formation. These biofilms were then scraped off the electrodes and used as inoculum for the formation of the three independent secondary biofilms used in this study. Growth of biofilms was monitored by measurements of the bioelectrocatalytic current production and by analysis of cyclic voltammetry profiles performed regularly. The media was regularly replenished with fresh growing media (generally once a week). The three secondary biofilms were incubated for a total of period of approximately 3 months. These biofilms were used for regular CRRM measurements as described below. All potentials provided in the manuscript are relative to the Ag/AgCl reference electrode (3 M KCl, 210 mV vs. the standard hydrogen electrode (SHE)) and the current density values are normalized versus the geometric (effective) surface area (2 cm^2^), unless otherwise indicated.

### Turnover and nonturnover cyclic voltammetry

Cyclic voltammetry tests under turnover conditions were performed with the biofilms immersed in their growing medium in the presence of 20 mM sodium acetate as metabolic electron donor. The potential of the working electrodes was swept between a lower and an upper limit (typically −0.7 V and 0 V) at a scan rate of 0.5 mV s^−1^. To perform nonturnover cyclic voltammetry tests (*i.e.*, in the absence of metabolic electron donor) the growing medium was first replenished with medium without acetate and the anode potential was set at 0 V until anodic oxidation current reached values below 1 µA cm^−2^ (typically after 12 hours). Cyclic voltammetry was performed by varying the potential of the working electrodes between −0.7 V and 0 V vs. Ag/AgCl. Scan rate analysis was conducted at increasing scan rates from 0.5 to 200 mV s^−1^.

### Confocal Raman measurements

To perform confocal Raman measurements, the anodes were removed from the incubation vessels and fixed into a three-electrode electrochemical apparatus (Figure S3) consisting of a sterile cell culture dish filled up with 50 mL of degassed autoclaved solution containing culturing media (*vide supra*), and equipped with a Ag/AgCl reference electrode and a platinum wire as counter electrode to allow for electrochemical measurements using a VMP3 potentiostat. The bioelectrochemical cell was then placed on the microscope stage for CRRM measurements. These were performed at room temperature (22±1°C) using an Alpha 300 Raman/AFM (WITek GmbH, Ulm, Germany) equipped with a frequency-doubled continuous-wave Nd:YAG laser to obtain a 532 nm excitation line. The laser beam was focused by an objective lens (Nikon 60X, N.A. 1, water immersion objective). The back-scattered Raman light from the sample was collected with a 50 µm optical fiber with a resolution of 4 cm^−1^. All measurements were conducted with a laser power <5 mW. Typically, spectral acquisition was done at integration times of 0.2 s, which provided to be sufficient to obtain high-contrast resonance spectra of the sample. Biofilm sectional images have been constructed by collecting spectra on 100 points/line with 1 µm step in depth and a scan line width of 60 µm. WITec Project Data Evaluation Software (WITec GmbH, Ulm, Germany) was used for spectra processing and images reconstruction.

Raman measurements on biofilms under potentiostatic conditions were conducted by applying a fixed potential to the working electrode (generally −0.6 V or 0 V vs. Ag/AgCl), in the presence or in the absence of acetate, as discussed below. The potentials were maintained for at least 20 minutes to allow for electrochemical stabilization of the biofilms before spectra acquisition, confirmed by stabilization of the current profile. Raman measurements on biofilms during cyclic voltammetry were performed using a potential window between −0.7 V and +0.7 V vs. Ag/AgCl and a scan rate of 100 mV s^−1^ whilst spectral information were continuously collected from a single biofilm spot at approximately 50 µm distance from the electrode. Time-series Raman traces were generated by plotting the variation of the spectral intensity of the Raman scattering binned for the bands at 750 cm^−1^ (*ν*_15_) and 1640 cm^−1^ (*ν*_10_), characteristic of the redox shifts of cytochromes.

## Supporting Information

Table S1 **Band assignment of prominent bands in the RR spectra of mixed-culture electroactive biofilms.** Normal mode assignment and allocation of local coordinates of the most prominent bands from the averaged Raman spectra obtained with the working electron poised at 0 or −0.6 V.
(PDF)

Figure S1 **Turnover CVs of electroactive biofilms recorded at different developing stages.** Turnover CVs recorded in the presence of 10 mM sodium acetate as metabolic substrate, A) at day 10, B) at day 57, C) at day 80.
(TIF)

Figure S2 **Profiles of current (red trace) and electrode potential (blue trace) recorded prior the collection of RR spectra from biofilms.** A) Nonturnover at 0 V (RR spectra reported in Figure 3C and D.) B) Nonturnover at −0.6 V (RR spectra reported in Figure 3G and H). C) Turnover at 0 V (RR spectra reported in Figure 5C and D).
(TIF)

Figure S3 **Schematic representation of the electrochemical cell used for confocal resonance Raman microscope observations.** A) Side view, and B) top view. WE: working electrode (glassy carbon). CE: counter electrode (Pt wire). RE: reference electrode (Ag/AgCl in 3 M KCl).
(TIF)
