# Supplementary material for: Real-Time Measurements of the Redox States of c-Type Cytochromes in Electroactive Biofilms: A Confocal Resonance Raman Microscopy Study
Source: PLoS One. 2014 Feb 25;9(2):e89918. doi: 10.1371/journal.pone.0089918 (PMC3934938; doi:10.1371/journal.pone.0089918)
Supplement: Table S1 — Band assignment of prominent bands in the RR spectra of mixed-culture electroactive biofilms. Normal mode assignment and allocation of local coordinates of the most prominent bands from the averaged Raman spectra obtained with the working electron poised at 0 or −0.6 V. (PDF) [file pone.0089918.s001.pdf]

Table S1. Normal mode assignment and allocation of local coordinates of the most prominent bands from the averaged Raman spectra obtained with the working electron poised at 0 or -0.6 V vs. Ag/AgCl.

**RR bands at -0.6 V (cm^{-1})**	**RR bands at 0 V (cm^{-1})**	**Assignment** [a]	**Local coordinates** [b]
751	753	ν_{15} (B_{1g})	ν(pyr breathing)
1132	1132	ν_{22} (A_{2g})	ν(pyr half-ring)$_{sym}$
1175	1173	ν_{30} (B_{2g})	ν(pyr half-ring)$_{sym}$
1230	1240	ν_{13} (B_{1g})	δ(CmH)
1316	1319	ν_{21} (A_{2g})	δ(CmH)
1366	1372	ν_4 (A_{1g})	ν(pyr half-ring)$_{sym}$
1396	1404	ν_{20} (A_{2g})	ν(pyr quarter-ring)
1500 (1492)	1506	ν_3 (A_{1g})	ν($C_\alpha C_m$)$_{sym}$
-	1564	ν_{11} (B_{1g})	ν($C_\beta C_\beta$)
1588	1588	ν_{19} (A_{2g}) / ν_2 (A_{1g})	ν($C_\alpha C_m$)$_{asym}$ / ν($C_\beta C_\beta$)
1640	1640	ν_{10} (B_{1g})	ν($C_\alpha C_m$)$_{asym}$

[a] assignment accordingly to Hu *et al.*[1]
[b] local coordinates accordingly to Johannessen *et al.*[2], and Li *et al.*[3]
Values in brackets are shoulders.

References

[1] S. Hu, I. Morris, J. Singh, K. Smith, T. G. Spiro, *J Am Chem Soc* **1993**, *115*, 12446–12458.
[2] C. Johannessen, P. C. White, S. Abdali, *J Phys Chem A* **2007**, *111*, 7771–7776.
[3] X. Y. Li, R. S. Czernuszewicz, J. R. Kincaid, Y. O. Su, T. G. Spiro, *The Journal of Physical Chemistry* **1990**, *94*, 31–47.
