# Supplementary figures and images for: Real-Time Measurements of the Redox States of c-Type Cytochromes in Electroactive Biofilms: A Confocal Resonance Raman Microscopy Study
Source: PLoS One. 2014 Feb 25;9(2):e89918. doi: 10.1371/journal.pone.0089918 (PMC3934938; doi:10.1371/journal.pone.0089918)

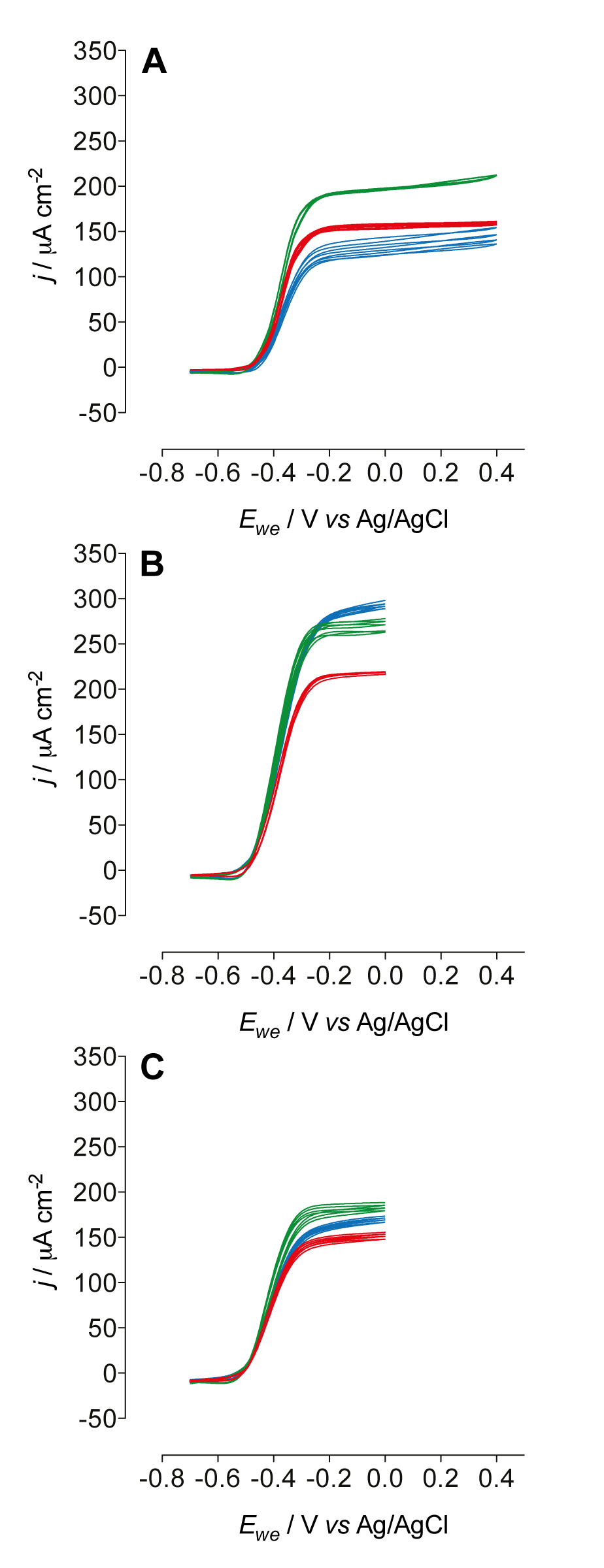

Supplement: Figure S1 — Turnover CVs of electroactive biofilms recorded at different developing stages. Turnover CVs recorded in the presence of 10 mM sodium acetate as metabolic substrate, A) at day 10, B) at day 57, C) at day 80. (TIF) [file pone.0089918.s002.tif]

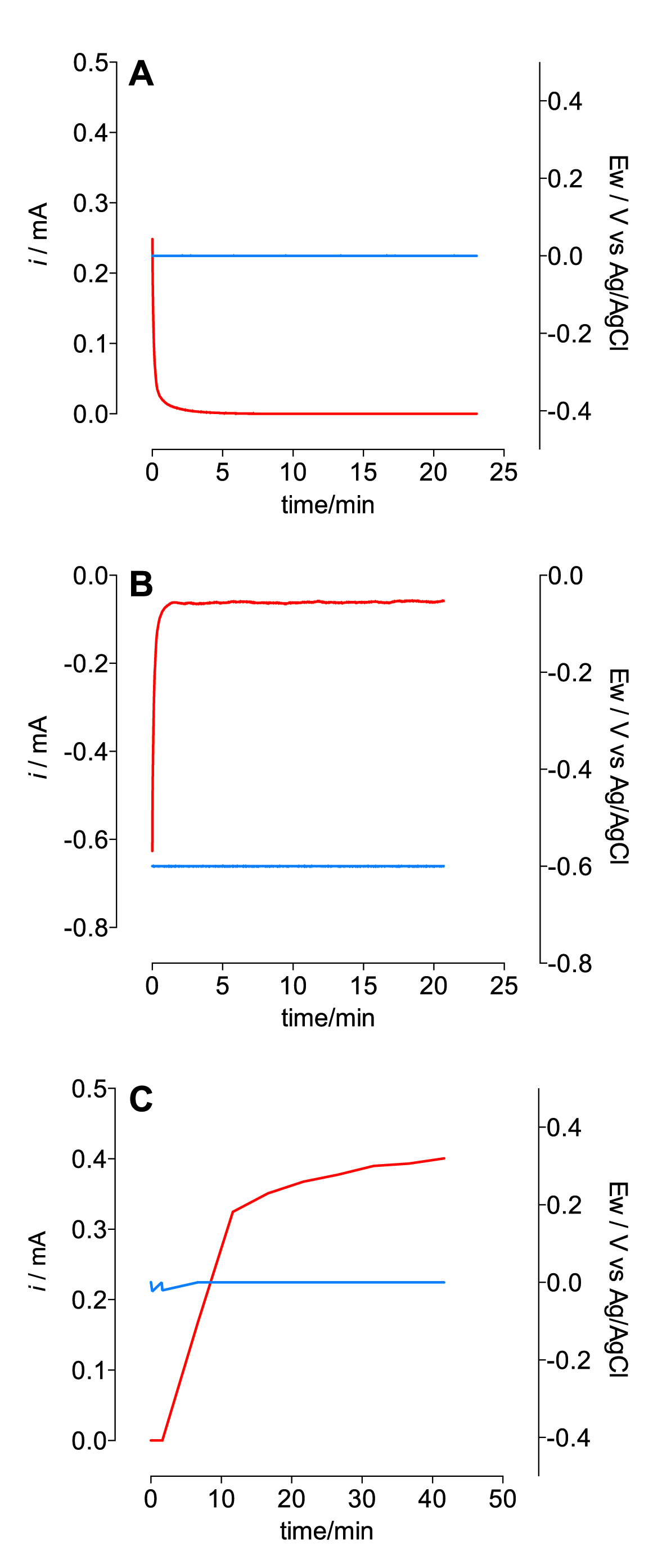

Supplement: Figure S2 — Profiles of current (red trace) and electrode potential (blue trace) recorded prior the collection of RR spectra from biofilms. A) Nonturnover at 0 V (RR spectra reported in Figure 3C and D.) B) Nonturnover at −0.6 V (RR spectra reported in Figure 3G and H). C) Turnover at 0 V (RR spectra reported in Figure 5C and D). (TIF) [file pone.0089918.s003.tif]

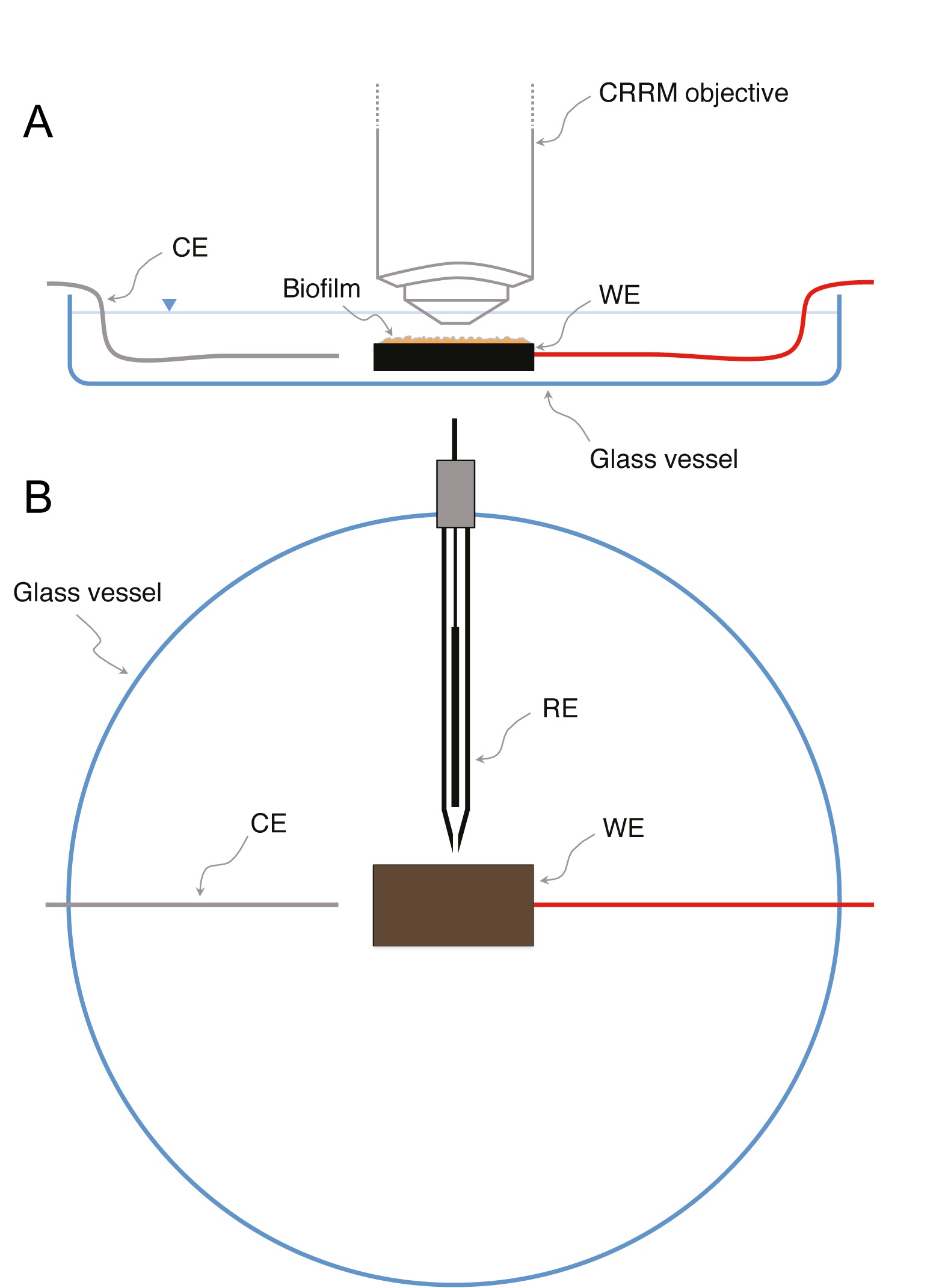

Supplement: Figure S3 — Schematic representation of the electrochemical cell used for confocal resonance Raman microscope observations. A) Side view, and B) top view. WE: working electrode (glassy carbon). CE: counter electrode (Pt wire). RE: reference electrode (Ag/AgCl in 3 M KCl). (TIF) [file pone.0089918.s004.tif]
